# Peripheral Opioid Receptor Blockade Enhances Epithelial Damage in Piroxicam-Accelerated Colitis in IL-10-Deficient Mice

**DOI:** 10.3390/ijms22147387

**Published:** 2021-07-09

**Authors:** Xavier Mas-Orea, Morgane Sebert, Mehdi Benamar, Camille Petitfils, Catherine Blanpied, Abdelhadi Saoudi, Céline Deraison, Frederick Barreau, Nicolas Cenac, Gilles Dietrich

**Affiliations:** 1 IRSD, Université de Toulouse—Paul Sabatier, INSERM, INRAe, ENVT, UPS, 31000 Toulouse, France; xavier.mas-orea@inserm.fr (X.M.-O.); morgane.sebert@hotmail.com (M.S.); camille.petitfils@inserm.fr (C.P.); catherine.blanpied@inserm.fr (C.B.); celine.deraison@inserm.fr (C.D.); frederick.barreau@inserm.fr (F.B.); nicolas.cenac@inserm.fr (N.C.); 2 INFINITY, Université de Toulouse—Paul Sabatier, INSERM, CNRS, UPS, 31000 Toulouse, France; Mehdi.Benamar@childrens.harvard.edu (M.B.); abdelhadi.saoudi@inserm.fr (A.S.)

**Keywords:** opioids, colitis, intestinal permeability, apoptosis, piroxicam, IL-10-deficient mice

## Abstract

Mucosal CD4^+^ T lymphocytes display a potent opioid-mediated analgesic activity in interleukin (IL)-10 knockout mouse model of inflammatory bowel diseases (IBD). Considering that endogenous opioids may also exhibit anti-inflammatory activities in the periphery, we examined the consequences of a peripheral opioid receptor blockade by naloxone-methiodide, a general opioid receptor antagonist unable to cross the blood–brain barrier, on the development of piroxicam-accelerated colitis in IL-10-deficient (IL-10^-/-^) mice. Here, we show that IL-10-deficient mice treated with piroxicam exhibited significant alterations of the intestinal barrier function, including permeability, inflammation-related bioactive lipid mediators, and mucosal CD4^+^ T lymphocyte subsets. Opioid receptor antagonization in the periphery had virtually no effect on colitis severity but significantly worsened epithelial cell apoptosis and intestinal permeability. Thus, although the endogenous opioid tone is not sufficient to reduce the severity of colitis significantly, it substantially contributes to the protection of the physical integrity of the epithelial barrier.

## 1. Introduction

Mucosal T lymphocytes play a key role in the endogenous regulation of visceral sensitivity in physiological as well as in pathological situations [1,2,3,4,5,6]. The analgesic effect of T lymphocytes is dependent on their ability to synthesize opioids upon antigen-priming and to release their opioid content at the vicinity of opioid receptors-expressing nociceptors [3,7,8,9,10,11,12]. Of note, even in the IL-10-deficient mouse model mimicking severe infantile Crohn’s disease associated with IL-10 loss of function, colitogenic CD4^+^ T lymphocytes conserve their analgesic activity [13]. Altogether these observations strengthen the rationale of a number of potential therapeutic strategies which propose to enhance or mimic immune-mediated endogenous opioid activity in chronic intestinal inflammatory diseases [14,15,16]. These therapeutic approaches aim at targeting opioid receptors in the periphery [17,18,19] and, for some of them, opioid receptors specifically located within the inflamed tissue [1,20,21,22,23,24] or at reinforcing endogenous immune-derived opioid tone [25,26]. Considering that opioid drugs also display anti-inflammatory effects [27,28,29,30], it may be expected that the alternative therapeutic strategies promoting mucosal endogenous opioid tone may also improve clinical outcomes in inflammatory bowel diseases (IBD).

Here, we examined the impact of opioids locally produced within the gut on the development of colitis in IL-10-deficient (IL-10^-/-^) mice. We used piroxicam, which has no effect on wild-type mice, to accelerate/synchronize the development of colitis in IL-10^-/-^ mice [31,32,33] and naloxone-methiodide, a general opioid receptor antagonist that does not cross the blood-brain barrier to neutralize opioid receptors in the periphery.

## 2. Results

### 2.1. Il-10^-/-^ Mice Treated with Piroxicam Develop Colitis

**Figure 1:**
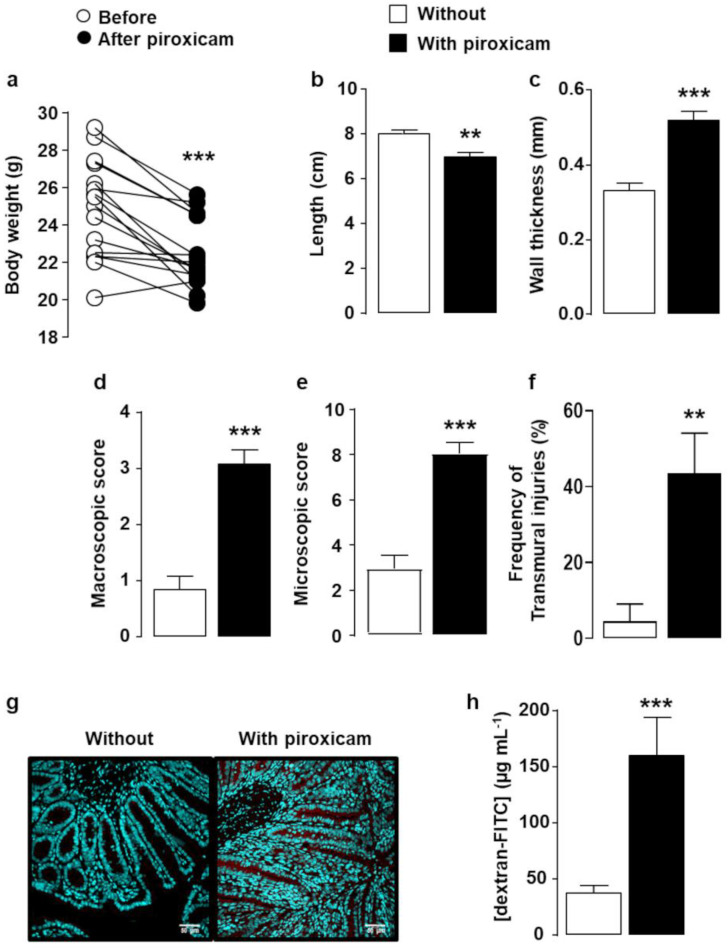
Colitis severity in IL-10^-/-^ mice fed with piroxicam. IL-10^-/-^ mice were fed with standard chow diet without (white) or with piroxicam (black) for 10 days (*n* = 14–18/group). Colitis parameters included body weight loss ((**a**), bodyweight before (white circle) and after piroxicam treatment (black circle), each symbol represents 1 mouse), colonic length (**b**), wall thickness (**c**), both macroscopic (**d**), and microscopic (**e**) colonic tissue damage including the percentage of transmural colon injuries (**f**). A representative immunohistological staining of apoptotic epithelial cells using anti-activated caspase 3 antibodies is shown in (**g**). Paracellular permeability expressed as the mean of FITC-dextran concentration in serum (µg mL^−1^) 4 h after gavage with dextran is depicted in (**h**). Data are expressed as mean ± SEM. Statistical analyses were performed using the Mann–Whitney U test except for panel A where statistical analysis was performed using the Wilcoxon matched pairs test, ** *p* < 0.01, *** *p* < 0.001.

IL-10^-/-^ mice fed with standard chow diet containing piroxicam for 10 days developed colitis characterized by a significant body weight loss (Figure 1a) and severe macroscopic (Figure 1b–d) and microscopic (Figure 1e,f) colonic lesions as compared to age-matched IL-10^-/-^ mice fed with standard chow diet free of piroxicam. Treatment with piroxicam induced the apoptosis of intestinal epithelial cells (virtually absent in untreated mice) as assessed by anti-active caspase 3 staining (Figure 1g), which, in turn, resulted in an increase in intestinal paracellular permeability as assessed by quantifying fluorescein isothiocyanate (FITC)-labeled dextran in blood four hours after administering it by gavage (Figure 1h).

**Figure 2:**
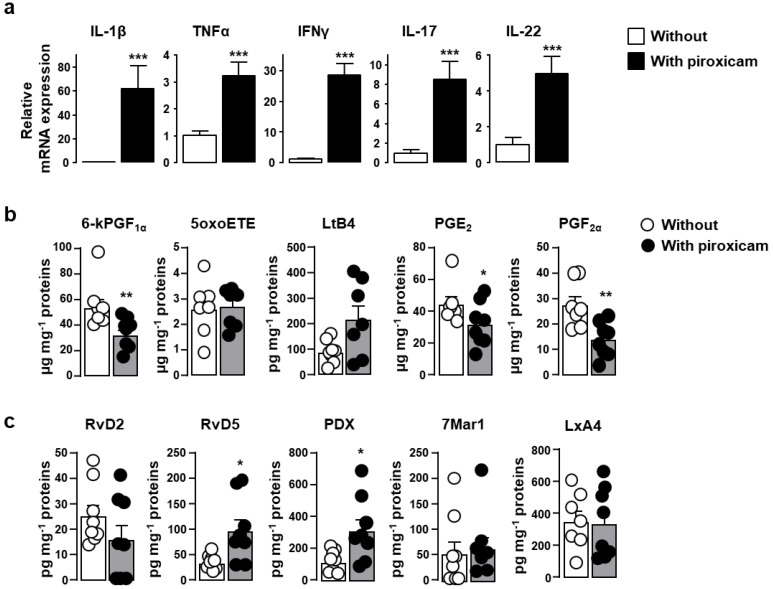
Inflammation-related parameters in IL-10^-/-^ mice fed with piroxicam. IL-10^-/-^ mice were fed with standard chow diet without (white symbols) or with piroxicam (black symbols) for 10 days. mRNA encoding for pro-inflammatory cytokines IL-1β, TNFα, IFNγ, IL-17, IL-22 was quantified by real-time PCR in colonic biopsies (**a**) (*n* = 14–18/group). mRNA content was normalized to the *hypoxanthine-guanine phosphoribosyltransferase* (*HPRT*) mRNA and quantified relative to standard cDNA prepared from referential mouse inflamed colonic tissue (calibrator samples). For each sample, mRNA level was expressed relative to the average of mRNA levels in untreated IL10^-/-^ mice. Data are expressed as mean ± SEM. Pro-inflammatory (**b**) and pro-resolving (**c**) bioactive lipids were quantified by liquid chromatography coupled to tandem mass spectrometry (LC-MS/MS) as expressed in pg or µg per milligrams of colonic tissue proteins (*n* = 8/group). Each symbol represents 1 mouse. Statistical analysis was performed using the Mann-Whitney U test. * *p* < 0.05, ** *p* < 0.01, *** *p* < 0.001.

The piroxicam-induced alteration of the epithelium integrity sufficient to favor bacterial translocation, elicits an immune response that, in the absence of the immunoregulatory activity of IL-10, leads to colitis [32]. Development of colitis was associated with a 3- to 60-fold increase in mRNA expression levels of pro-inflammatory cytokines including IL-1β, tumor necrosis factor (TNF)-α, interferon (IFN)-γ, IL-17, and IL-22 (Figure 2a). The tissue production of 5-lipoxygenase-derived pro-inflammatory lipids such as 5-oxoeicosatetraenoic acid (5-oxoETE) and leukotriene B4 (LtB_4_) remained unchanged while that of cyclooxygenase-derived lipids including 6-keto-prostaglandin F1 alpha (6-kPGF_1α_), PGE_2_ and PGF_2α_, was reduced (Figure 2b) in agreement with the inhibitory effect of piroxicam on the cyclooxygenase activity [31]. By contrast, two out of four docosahexaenoic acid (DHA)-derived pro-resolving lipids, including resolvin D5 (RvD5) and protectin Dx (PDx) were increased (Figure 2c).

**Figure 3:**
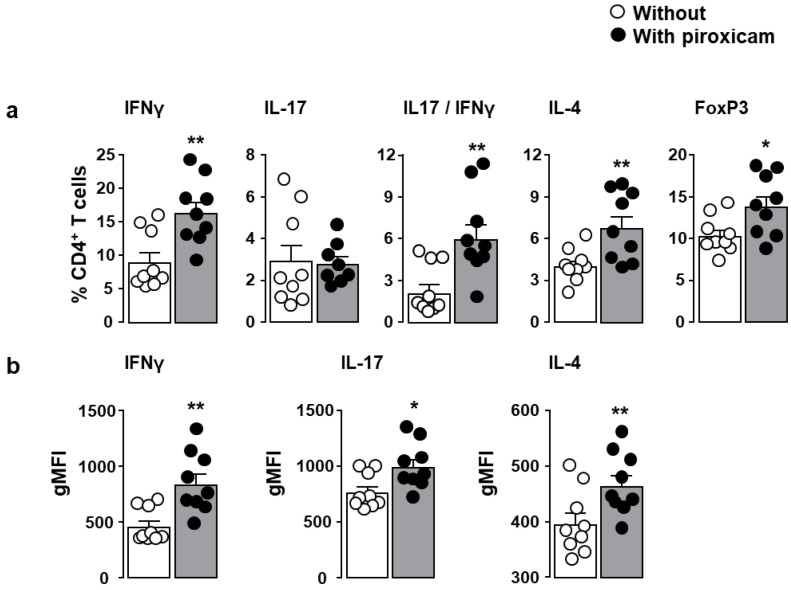
Frequency of CD4^+^ T lymphocytes with Th1, Th17, Th2, and Treg (regulatory T cells) phenotypes in IL-10^-/-^ mice fed with piroxicam. IL-10^-/-^ mice were fed with standard chow diet without (white circles) or with piroxicam (black circles) for 10 days. The frequencies of CD4^+^ T lymphocytes producing IFNγ (Th1), IL-17 (Th17), both IFNγ and IL-17 (Th17), or IL-4 (Th2), as well as CD4^+^ T lymphocytes expressing the lineage specification transcriptional factor Foxp3 (Treg) within *lamina propria*, were analyzed by cytofluorometry (**a**). The corresponding cytokine expression levels expressed as geometric mean of fluorescence intensity (gMFI) are depicted in panel (**b**). Each symbol represents 1 mouse (*n* = 9/group). Statistical analysis was performed using the Mann–Whitney U test. * *p* < 0.05, ** *p* < 0.01.

Colitis in piroxicam-treated IL-10^-/-^ mice was associated, within the *lamina propria,* with an increase in the frequencies of CD4^+^ T lymphocytes producing either IFNγ (Th1), both IFNγ and IL-17 (a feature of colitis-associated Th17) or IL-4 (Th2) as well as FoxP3-expressing regulatory T lymphocytes as compared to untreated IL-10^-/-^ mice (Figure 3a). Although the frequency of single IL-17 producers was unchanged, the increase in the other CD4^+^ T lymphocyte subsets correlated with increased production of the relevant cytokines IFNγ (Th1), IL-17 (Th17) and IL-4 (Th2) as assessed by geometric mean of fluorescence intensity (gMFI) (Figure 3b). The number of CD4^+^ T lymphocytes within the *lamina propria* was similar (data not shown).

### 2.2. Peripheral Opioid Receptor Blockade by Naloxone-Methiodide Increases Intestinal Epithelial Cell Apoptosis in Il-10^-/-^ Mice with Colitis

**Figure 4:**
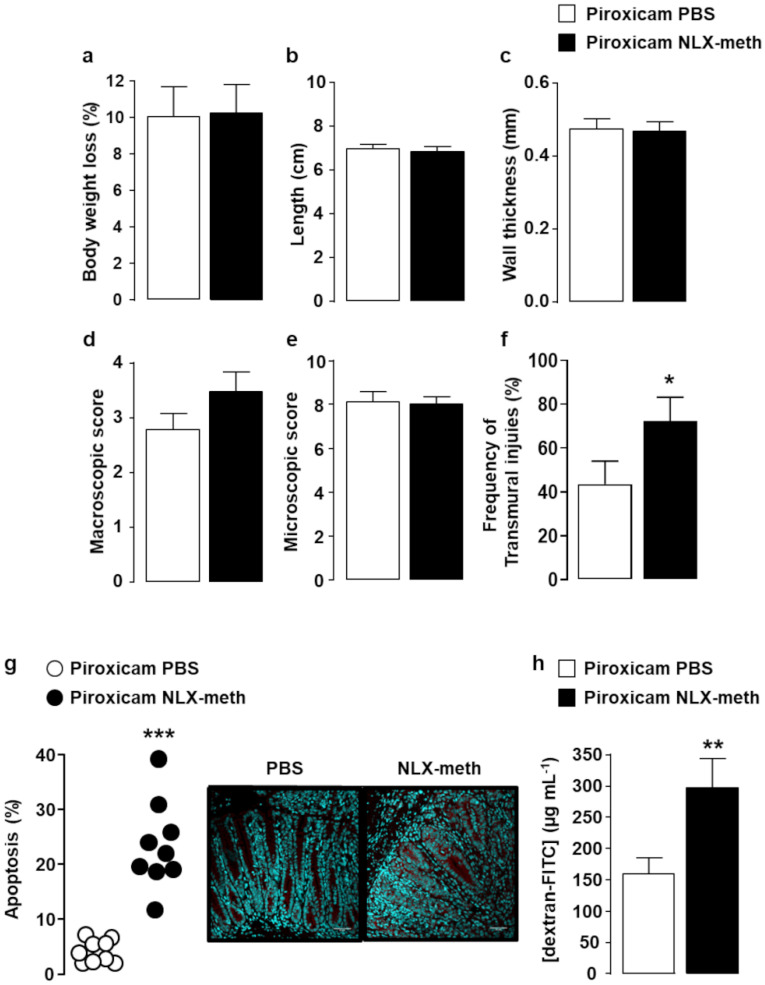
Effects of peripheral opioid receptor blockade on colitis severity in piroxicam-treated IL-10^-/-^ mice. IL10^-/-^ mice were injected with PBS (white symbols) or naloxone-methiodide (NLX-meth, 2 mg) (black symbols) two days apart for the ten days of piroxicam treatment (*n* = 17–20/group). Colitis parameters included percent of body weight loss (**a**), colonic length (**b**), wall thickness (**c**), both macroscopic (**d**) and microscopic (**e**) colonic tissue damage including the percentage of transmural colon injuries (**f**). Epithelial apoptosis identified with anti-activated caspase 3 antibodies was quantified relative to epithelium surface ((**g**) left panel, each symbol represents 1 mouse). A representative immunohistological staining of apoptotic epithelial cells is shown in the right panel. Paracellular permeability expressed as the mean of FITC-dextran concentration in serum (µg mL^−1^) 4 h after gavage with dextran (*n* = 10–13/group) is depicted in (**h**). Data are expressed as mean ± SEM. Statistical analysis was performed using the Mann-Whitney U test. * *p* < 0.05, ** *p* < 0.01, *** *p* < 0.001.

In order to examine the impact of the mucosal opioid tone on the development of colitis, IL-10^-/-^ mice fed with standard chow diet containing piroxicam were intraperitoneally injected over the 10 days of treatment with either PBS or naloxone-methiodide (NLX-methiodide), a general antagonist of opioid receptors unable to cross the blood-brain barrier, that has no pro-inflammatory effect *per se* in normal mice (Supplementary Figure S1). As shown in Figure 4, although the frequency of transmural colonic damage was increased (Figure 4f), NLX-methiodide treatment did not worsen the colitis as assessed by body weight loss (Figure 4a), colonic length (Figure 4b), colonic wall thickening (Figure 4c), macroscopic and microscopic colonic lesions (Figure 4d,e). However, even if the severity of colitis remained virtually unchanged, peripheral opioid receptor neutralization resulted in an increase in both epithelial cell apoptosis (Figure 4g) and intestinal permeability (Figure 4h).

**Figure 5:**
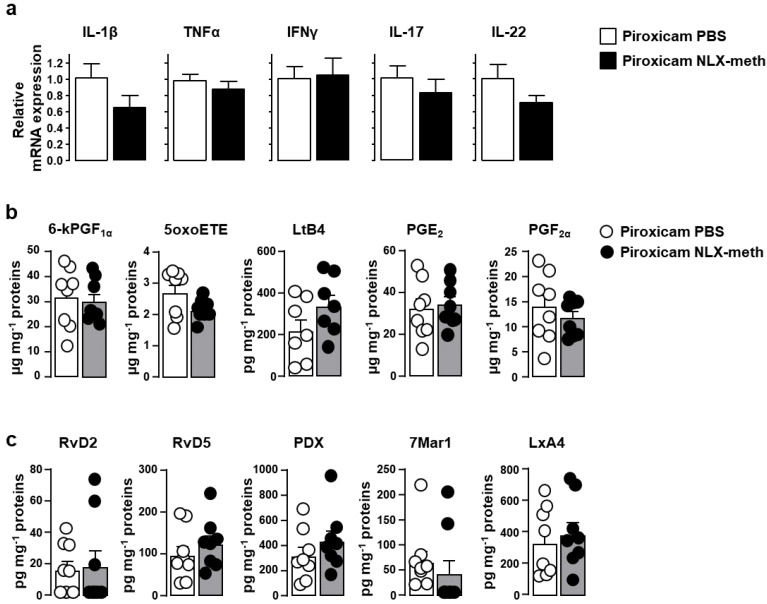
Effects of peripheral opioid receptor blockade on inflammation-related parameters in piroxicam-treated IL-10^-/-^. IL10^-/-^ mice were injected with PBS (white symbols) or naloxone-methiodide (NLX-meth) (black symbols) two days apart for the ten days of piroxicam treatment. mRNA encoding for pro-inflammatory cytokines IL-1β, TNFα, IFNγ, IL-17, IL-22 was quantified by real-time PCR in colonic biopsies (**a**) (*n* = 13–17/group) as described above. Data are expressed as mean ± SEM. Pro-inflammatory (**b**) and pro-resolving (**c**) bioactive lipids were quantified by LC-MS/MS as expressed in pg or µg per milligrams of colonic tissue proteins (*n* = 8/group). Each symbol represents 1 mouse. Statistical analysis was performed using the Mann-Whitney U test.

mRNA expression levels of all the pro-inflammatory cytokines were unchanged following peripheral opioid receptor antagonization by naloxone-methiodide (Figure 5a). Similarly, no effect was observed on tissue production of all the polyunsaturated fatty acids with pro-inflammatory (Figure 5b) or pro-resolving (Figure 5c) activities that we examined.

**Figure 6:**
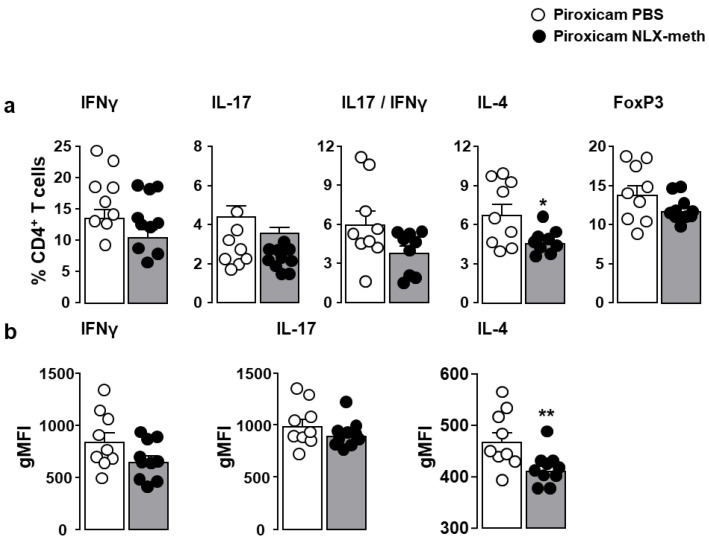
Effects of peripheral opioid receptor blockade on the frequency of Th1, Th17, Th2, and Treg in piroxicam-treated IL-10^-/-^. IL10^-/-^ mice were injected with PBS (white circles) or naloxone-methiodide (NLX-meth) (black circles) two days apart for the ten days of piroxicam treatment. The frequencies of CD4^+^ T lymphocytes producing IFNγ (Th1), IL-17 (Th17), both IFNγ and IL-17 (Th17), or IL-4 (Th2) as well as CD4^+^ T lymphocytes expressing the lineage specification transcriptional factor Foxp3 (Treg) within *lamina propria* were analyzed by cytofluorometry (**a**). The corresponding cytokine expression levels expressed as the geometric mean of fluorescence intensity (gMFI) are depicted in panel (**b**). Each symbol represents 1 mouse (*n* = 9–10/group). Statistical analysis was performed using the Mann–Whitney U test. * *p* < 0.05, ** *p* < 0.01.

The frequency of Th1, Th17, and FoxP3^+^ Treg subsets of mucosal CD4^+^ T lymphocytes as well as their respective ability to produce the prototypical cytokines IFNγ and/or IL-17 was not affected by the neutralization of opioid receptors in the periphery. Contrasting with the Th1 and Th17 subsets, the frequency of Th2 lymphocytes as well as the expression level of IL-4 was significantly reduced by naloxone-methiodide treatment (Figure 6a,b). The number of CD4^+^ T lymphocytes within the *lamina propria* was unchanged (data not shown).

## 3. Discussion

Anomalies in genes related to the immune response and/or the physical intestinal barrier are predisposing to intestinal bowel diseases (IBD) such as Crohn’s disease [34]. Among immune-related genes associated with Crohn’s disease, those involving the IL-10 regulatory pathway are associated with severe forms of the disease [35,36,37]. In IL-10^-/-^ mice, the spontaneous onset of the colitis has been associated with an increased intestinal permeability [38], a primary event mimicked by the exposure to piroxicam, a non-steroidal anti-inflammatory drug inducing epithelial cell apoptosis [32]. The increase in intestinal permeability favors the passage of bacteria from the lumen towards the *lamina propria*, thereby inducing an immune response that, in the absence of efficient regulation, results in chronic inflammation [32,33,39]. As expected, piroxicam-induced colitis in IL-10^-/-^ mice was associated with a huge increase in inflammatory cytokines within the inflamed colonic tissue. Th1, Th17, and Th2 subsets of mucosal CD4^+^ T lymphocytes were increased, but the frequency of Th1 and Th17 colitogenic T cells were nearly 4-fold-higher than that of Th2. As reported in IBD patients, the frequency of FoxP3^+^ Treg was increased, but genetic deletion of IL-10 makes them not efficient enough [40,41,42,43,44]. In IL-10^-/-^ mice, as for a number of other models including Th1/Th17- or Th2-driven colitis, colitogenic T lymphocytes maintain their ability to produce enkephalins [2,4,13,45].

Immune-derived opioids exhibit anti-inflammatory activities in Th1/Th17-driven colitis because of their propensity to favor the commitment of lymphocytes towards a Th2 phenotype [45,46,47,48,49]. However, the anti-inflammatory effects of endogenous opioids have been shown in colitis models mimicking an excessive immune response in which innate and adaptive immune cells display unaltered functional properties [1,2,27,28,45]. Moreover, although the reduction of the inflammatory cell activity is primordial, the restoration of the epithelial barrier integrity is necessary to minimize bacterial-induced inflammatory response [50,51,52]. Accordingly, we investigated the endogenous opioid-mediated regulation of peripheral inflammation and epithelial barrier integrity in IBD-like colitis in IL-10^-/-^ mice. Colitis severity and inflammation parameters, including pro-inflammatory cytokines and bioactive lipids, remained unchanged following naloxone-methiodide-mediated neutralization of peripheral opioid receptors. The frequency of colitogenic Th1/Th17 lymphocytes and Treg was not affected by naloxone-methiodide treatment. In agreement with the Th2-promoting activity of endogenous opioids, piroxicam-treated IL-10^-/-^ mice injected with naloxone-methiodide displayed a significant decrease in mucosal IL-4-producing Th2 lymphocyte frequency.

In line with previous results reporting a beneficial effect of opioids on both intestinal permeability and epithelium recovery [53,54,55], our study shows that neutralization of endogenous opioid activity in the periphery results in an increase in epithelial cell apoptosis and permeability. Although the pleiotropic distribution of opioid receptors within the intestinal tract allows a number of alternatives to explain the alterations of intestinal permeability by naloxone-methiodide treatment, two hypotheses may be proposed. Bacteria that cross the epithelial barrier to enter into intestinal mucosa may activate, among others, mast cells through toll-like receptors (TLR) and/or N-formyl-methionyl-leucyl-phenylalanine (fmlp) receptors [56,57]. In turn, inflammatory mediators released by activated mast cells worsen intestinal epithelial barrier dysfunction and thereby increase intestinal barrier permeability [58,59]. Considering that both TLR and fmlp signaling pathways may be inhibited by opioids [29,58,60], the increase in intestinal permeability observed in naloxone-methiodide-treated mice may be dependent on the neutralization of the opioid effect on mast cell activity. Alternatively, CD95 (Fas/APO-1), constitutively expressed at the basolateral side of intestinal epithelial cells [61], has been reported as protecting the epithelium in colitis [62]. Thus, although CD95-mediated signaling is usually associated with apoptosis, the specific deletion of the CD95 gene or CD95 signaling deficiency in the intestinal epithelium worsens inflammation-induced colonic injury [62]. The targeted deletion of CD95 in intestinal epithelial cells, without any alteration of CD95 expression in immune cells, results in a significant delay in the mucosal regeneration [62]. Given that endogenous opioids may up-regulate CD95 (Fas/APO-1) expression on the cell surface [63,64,65], it could be speculated that naloxone-methiodide-increased permeability may be dependent on a reduction of CD95 (Fas/APO-1) expression in the intestinal epithelium.

## 4. Conclusions

Taken together, our study shows that the novel therapeutic strategies, which propose to harness or mimic the mucosal endogenous opioid tone to relieve inflammatory bowel pain, may also be instrumental in improving inflammation-induced colonic epithelial damage.

## 5. Materials and Methods

### 5.1. Mice and Naloxone-Methiodide (NLX-Meth) Administration

Eight- to twelve-week-old *IL-10* gene-deficient male (IL-10^-/-^) mice on C57BL/6 genetic background were bred in the animal care facility at Toulouse (INSERM US 006 ANEXPLO/CREFRE, Toulouse, France). Colitis was accelerated/synchronized by adding 150 mg kg^−1^ piroxicam, a non-steroidal anti-inflammatory drug, into standard chow diet for 10 days (SAFE, Scientific Animal Food & Engineering, Rosenberg, Germany) [13]. Mice were intraperitoneally injected with 200 µL of either PBS or 10 mg mL^−1^ naloxone-methiodide (Sigma Chemical Co., St. Louis, MO, USA) (2 mg/mouse) two days apart for the ten days of piroxicam treatment [63,66].

### 5.2. Macroscopic Assessment of Colonic Injury

Macroscopic colonic tissue damage was evaluated using a scale ranging from 0 to 11 as follows: erythema (absent (0), length of the area < 1 cm (1), more than 1 cm (2)), edema (absent (0), mild (1), severe (2)), strictures (absent (0), one (1), two (2), more than two (3)), ulceration (absent (0), present (1)), mucus (present (0), absent (1)), and adhesion (absent (0), moderate (1), severe (2)). Bowel wall thickness was measured with an electronic caliper in the distal part of the colon, at 0.5 cm above the anus.

### 5.3. Histological Assessment of Colonic Injury

Colonic tissue specimens were excised 2 cm proximal to the anus and immediately transferred into 4% paraformaldehyde to be embedded in paraffin. Five-micrometer colonic sections were then stained with hematoxylin-eosin (HE). Damage scoring was evaluated on a scale ranging from 0 to 12. Inflammatory cell infiltration, epithelial/mucosal alteration (including vasculitis, goblet cell depletion, and crypt abscesses), mucosal architecture alteration (including ulceration and crypt loss), and submucosal edema were graded from 0 to 3 (absent, mild, moderate, and severe) [1].

### 5.4. Intestinal Epithelial Cell Apoptosis Assessment

Epithelial cell apoptosis was quantified by immunohistochemical staining of activated caspase-3 in colonic biopsies embedded in paraffin (colonic specimen were the same as described above). Slices were incubated with PBS containing 3% bovine serum albumin (BSA, Sigma) and 2.5% normal donkey serum (Sigma) for 90 min at room temperature (RT). Slices were then incubated with rabbit anti-activated caspase 3 polyclonal IgG antibodies (Abcam ab13847, Cambridge, UK, 1/200) for 90 min at RT. After extensive washing with PBS, bound antibodies were revealed by adding AlexaFluor 555-conjugated donkey anti-rabbit IgG (Life Technologies, Carlsbad, CA, USA, 1/500) for an additional 90 min period of time at RT. Nuclei were then stained with DAPI for 20 min (Invitrogen, Carlsbad, CA, USA) and the slices were mounted with ProLong Gold mounting medium (LifeTechnologies). Images were acquired with Zeiss 710 inverted confocal microscope (×20 NA 0.8). Epithelial apoptosis rate was calculated by measuring the area of activated caspase 3-expressing cells relative to the total area of the epithelial cells (inverted threshold 200 and analyze particles) [63].

### 5.5. Real-Time Quantitative PCR Analysis

Colonic tissue samples were homogenized in 500 µL TRIzol™ Reagent (Sigma). Total RNA was then isolated by using GenElute™ Mammalian total RNA miniprep Kit following the manufacturer’s instructions (Sigma). RNA was reverse-transcribed with Moloney murine leukemia virus reverse transcriptase using random hexamers for priming. Transcripts encoding hypoxanthine phosphoribosyl transferase (HPRT), TNFα, IFNγ, IL-1β, IL-17, IL-22 were quantified by real-time quantitative polymerase chain reaction using the following forward and reverse primers: 5′-GTTCTTTGCTGACCTGCTGGAT-3′ and 5′-CCCCGTTGACTGATCATTACAG-3′ for HPRT, 5′-CCACGCTCTTCTGTCTACTGAAC-3′ and 5′-GGTCTGGGCCATAGAACTGATG-3′ for TNFα, 5′-CAGCAACAGCAAGGCGAAA-3′ and 5′-AGCTCATTGAATGCTTGGCG-3′ for IFNγ, 5′-ACCTTCCAGGATGAGGACATGAG-3′ and 5′-CATCCCATGAGTCACAGAGGATG-3′ for IL-1β, 5′-TCCAGAAGGCCCTCAGACTA-3′ and 5′-CAGGATCTCTTGCTGGATG-3′ for IL-17, 5′-ACCGCTGATGTGACAGGAGC-3′ and 5′-AGGTGGTGCCTTTCCTGACC-3′ for IL-22. The target gene expression was normalized to the HPRT mRNA and quantified relative to a standard cDNA (calibrator sample) prepared from mouse inflamed colonic tissue using the 2^−ΔΔ*C*T^ method, where ΔΔ*C*_T_ = Δ*C*_T sample_ − Δ*C*_T calibrator_ [13,67]. For each sample, mRNA level was then expressed relative to the average of mRNA levels in untreated IL10^-/-^ mice.

### 5.6. Assessment of Intestinal Paracellular Permeability In Vivo

In vivo, intestinal permeability was assessed by gavaging mice with 12 mg of 4 kDa FITC-dextran (Sigma), a tracer of paracellular permeability. Four hours after gavage, FITC-dextran was quantified in the serum by measuring fluorescence intensity using an automatic Infinite M200 microplate reader (Tecan, Männedorf Switzerland). Concentrations were calculated from standard curves of 4 kDa FITC-dextran [68].

### 5.7. Isolation of Lamina Propria Mononuclear Cells

The intestine was longitudinally opened, cut into small pieces, washed, and incubated twice with RPMI 5% FCS 5 mM EDTA at 37 °C for 15 min. After washing, colonic tissues were digested with 0.02% collagenase VIII (Sigma) for 1 h at 37 °C. The supernatant was then passed through a 70 µm cell strainer and centrifuged. Mononuclear cells were then isolated upon Percoll gradient [2].

### 5.8. Cytofluorometric Analysis

Mononuclear cells purified from *lamina propria* were stimulated with 0.5 µg mL^−1^ phorbol myristate acetate and 1 µg mL^−1^ ionomycin (Sigma) in the presence of the protein transport inhibitor Brefeldin A (1 μg mL^−1^, GolgiPlug, BD Biosciences, Franklin Lakes, NJ, USA) for 4 h. After washing, the cells were first incubated with blocking buffer (PBS with 1% FCS, 3% normal mouse serum, 3% normal rat serum, 5 mM EDTA, 0.1% NaN_3_) containing 5 µg mL^−1^ rat anti-CD16/CD32 mAb (mouse Fc block^TM^, clone 2.4G2, BD Biosciences) for 15 min at room temperature. Cells were incubated with Viability Dye eFluor™ 780 (e-bioscience, Thermo Fisher Scientific, Waltham, MA, USA), and CD4^+^ T lymphocytes were stained with both BV510-conjugated rat anti-mouse CD4 (clone RM4-5) and PE-Cy7-conjugated hamster anti-mouse TCR-β chain (clone H57-597) mAbs diluted at the optimal concentration in FACS buffer (PBS 1% FCS, 5 mM EDTA, 0.1% NaN_3_) for 30 min on ice. Cells were then fixed with 2% paraformaldehyde in PBS and permeabilized using BD Cytofix/Cytoperm solution (e-Biosciences) according to the manufacturer’s instructions. Intracellular cytokine staining was then performed with Alexa Fluor^®^ 488-conjugated rat anti-IFN-γ (clone XMG1.2), Alexa Fluor^®^ 700-conjugated rat anti-IL-17 (clone TC11-18H10.1), eFluor 450-conjugated rat anti-Foxp3 (clone FJK-16s) and phycoerythrin-conjugated rat anti-IL-4 (clone 11B11) mAbs for 20 min and washed twice with Perm/Wash buffer before analysis. Specific anti-cytokine antibodies and isotype-matched control antibodies were purchased from e-Bioscience, BD Biosciences, and BioLegend (San Diego, CA, USA). Data were acquired on 100,000 cells by forward and side scatter intensity on a Fortessa (BD Biosciences) and further analyzed using the FlowJo software (Tree Star, Ashland, OR, USA) [45].

### 5.9. Quantification of Polyunsaturated Fatty Acids (PUFA) and Their Metabolites

Colonic biopsies were crushed with a FastPrep-24 Instrument (MP Biomedical, Fisher Scientific SAS, Illkirch, France) in 200 μL HBSS (Invitrogen) and 5 μL internal standard mixture (Deuterium-labeled compounds) (400 ng mL^−1^). After two crush cycles (6.5 m s^−1^, 30 s), 10 μL of suspensions were withdrawn for protein quantification and 0.3 mL of cold methanol (MeOH) were added. Samples were centrifuged at 1016× *g* for 15 min and supernatants were then submitted to solid-phase extraction of lipids using HLB plate (OASIS^®^ HLB mg, 96-well plate, Waters, Saint-Quentin-en-Yvelines, France). At the end of the process, samples were suspended in 10 µL of methanol for liquid chromatography/mass spectrometry analysis. 6-keto-prostaglandin F1 alpha (6-kPGF_1α_), PGE_2_, PGF_2α_, lipoxin A4 (LxA_4_), resolvin D2 (RvD2), RvD5, 7-Maresin 1 (7-Mar1), leukotriene B4 (LtB_4_), protectin Dx (PDx), and 5-oxoeicosatetraenoic acid (5-oxoETE) were quantified by an ultrahigh-performance liquid chromatography system (UHPLC; Agilent LC1290 Infinity) coupled to an Agilent 6460 triple quadrupole MS (Agilent Technologies, Santa Clara, CA, USA) equipped with electrospray ionization operating in negative mode. Data were acquired in multiple reaction monitoring (MRM) mode with optimized conditions. Peak detection, integration, and quantitative analysis were performed with MassHunter Quantitative analysis software (Agilent Technologies, Santa Clara, CA, USA). For each standard, calibration curves were built using 10 solutions at concentrations ranging from 0.95 to 500 ng mL^−1^ [69,70].

## Data Availability

The data that support the findings of this study are available from the corresponding author upon reasonable request.

## Supplementary Materials

The following are available online at https://www.mdpi.com/article/10.3390/ijms22147387/s1, Figure S1: Effects of peripheral opioid receptor blockade on colon of normal mice.

## References

[1] Basso L., Boue J., Auge C., Deraison C., Blanpied C., Cenac N., Lluel P., Vergnolle N., Dietrich G. (2018). Mobilization of CD4^+^ T lymphocytes in inflamed mucosa reduces pain in colitis mice: Toward a vaccinal strategy to alleviate inflammatory visceral pain. Pain.

[2] Boue J., Basso L., Cenac N., Blanpied C., Rolli-Derkinderen M., Neunlist M., Vergnolle N., Dietrich G. (2014). Endogenous regulation of visceral pain via production of opioids by colitogenic CD4^+^ T cells in mice. Gastroenterology.

[3] Hughes P.A., Harrington A.M., Castro J., Liebregts T., Adam B., Grasby D.J., Isaacs N.J., Maldeniya L., Martin C.M., Persson J. (2013). Sensory neuro-immune interactions differ between irritable bowel syndrome subtypes. Gut.

[4] Valdez-Morales E., Guerrero-Alba R., Ochoa-Cortes F., Benson J., Spreadbury I., Hurlbut D., Miranda-Morales M., Lomax A.E., Vanner S. (2013). Release of endogenous opioids during a chronic IBD model suppresses the excitability of colonic DRG neurons. Neurogastroenterol. Motil..

[5] Verma-Gandhu M., Bercik P., Motomura Y., Verdu E.F., Khan W.I., Blennerhassett P.A., Wang L., El-Sharkawy R.T., Collins S.M. (2006). CD4^+^ T-cell modulation of visceral nociception in mice. Gastroenterology.

[6] Verma-Gandhu M., Verdu E.F., Bercik P., Blennerhassett P.A., Al-Mutawaly N., Ghia J.E., Collins S.M. (2007). Visceral pain perception is determined by the duration of colitis and associated neuropeptide expression in the mouse. Gut.

[7] Boue J., Blanpied C., Brousset P., Vergnolle N., Dietrich G. (2011). Endogenous opioid-mediated analgesia is dependent on adaptive T cell response in mice. J. Immunol..

[8] Boue J., Blanpied C., Djata-Cabral M., Pelletier L., Vergnolle N., Dietrich G. (2012). Immune conditions associated with CD4^+^ T effector-induced opioid release and analgesia. Pain.

[9] Brack A., Rittner H.L., Machelska H., Shaqura M., Mousa S.A., Labuz D., Zollner C., Schafer M., Stein C. (2004). Endogenous peripheral antinociception in early inflammation is not limited by the number of opioid-containing leukocytes but by opioid receptor expression. Pain.

[10] Guerrero-Alba R., Valdez-Morales E.E., Jimenez-Vargas N.N., Bron R., Poole D., Reed D., Castro J., Campaniello M., Hughes P.A., Brierley S.M. (2018). Co-expression of mu and delta opioid receptors by mouse colonic nociceptors. Br. J. Pharmacol..

[11] Machelska H., Celik M.O. (2020). Immune cell-mediated opioid analgesia. Immunol. Lett..

[12] Stein C., Schafer M., Machelska H. (2003). Attacking pain at its source: New perspectives on opioids. Nat. Med..

[13] Basso L., Benamar M., Mas-Orea X., Deraison C., Blanpied C., Cenac N., Saoudi A., Dietrich G. (2020). Endogenous control of inflammatory visceral pain by T cell-derived opioids in IL-10-deficient mice. Neurogastroenterol. Motil..

[14] Basso L., Bourreille A., Dietrich G. (2015). Intestinal inflammation and pain management. Curr. Opin. Pharmacol..

[15] Carbone S.E., Poole D.P. (2020). Inflammation without pain: Immune-derived opioids hold the key. Neurogastroenterol. Motil..

[16] Stein C. (2018). New concepts in opioid analgesia. Expert Opin. Investig. Drugs.

[17] Feng J., Lepetre-Mouelhi S., Gautier A., Mura S., Cailleau C., Coudore F., Hamon M., Couvreur P. (2019). A new painkiller nanomedicine to bypass the blood-brain barrier and the use of morphine. Sci. Adv..

[18] Mace G., Blanpied C., Emorine L.J., Druet P., Dietrich G. (1999). Morphine-like activity of natural human IgG autoantibodies is because of binding to the first and third extracellular loops of the mu-opioid receptor. J. Biol. Chem..

[19] Binning A.R., Przesmycki K., Sowinski P., Morrison L.M., Smith T.W., Marcus P., Lees J.P., Dahan A. (2011). A randomised controlled trial on the efficacy and side-effect profile (nausea/vomiting/sedation) of morphine-6-glucuronide versus morphine for post-operative pain relief after major abdominal surgery. Eur. J. Pain.

[20] Hua S., Cabot P.J. (2013). Targeted nanoparticles that mimic immune cells in pain control inducing analgesic and anti-inflammatory actions: A potential novel treatment of acute and chronic pain condition. Pain Physician.

[21] Rodriguez-Gaztelumendi A., Spahn V., Labuz D., Machelska H., Stein C. (2018). Analgesic effects of a novel pH-dependent mu-opioid receptor agonist in models of neuropathic and abdominal pain. Pain.

[22] Spahn V., Del Vecchio G., Labuz D., Rodriguez-Gaztelumendi A., Massaly N., Temp J., Durmaz V., Sabri P., Reidelbach M., Machelska H. (2017). A nontoxic pain killer designed by modeling of pathological receptor conformations. Science.

[23] Jimenez-Vargas N.N., Yu Y., Jensen D.D., Bok D.D., Wisdom M., Latorre R., Lopez C., Jaramillo-Polanco J.O., Degro C., Guzman-Rodriguez M. (2021). Agonist that activates the micro-opioid receptor in acidified microenvironments inhibits colitis pain without side effects. Gut.

[24] Auge C., Basso L., Blanpied C., Vergnolle N., Game X., Chabot S., Lluel P., Dietrich G. (2021). Pain management in a model of Interstitial Cystitis/Bladder Pain Syndrome by a vaccinal strategy. Front. Pain Res..

[25] Burford N.T., Traynor J.R., Alt A. (2015). Positive allosteric modulators of the mu-opioid receptor: A novel approach for future pain medications. Br. J. Pharmacol..

[26] Reaux-Le Goazigo A., Poras H., Ben-Dhaou C., Ouimet T., Baudouin C., Wurm M., Melik Parsadaniantz S. (2019). Dual enkephalinase inhibitor PL265: A novel topical treatment to alleviate corneal pain and inflammation. Pain.

[27] Anselmi L., Huynh J., Duraffourd C., Jaramillo I., Vegezzi G., Saccani F., Boschetti E., Brecha N.C., De Giorgio R., Sternini C. (2015). Activation of mu opioid receptors modulates inflammation in acute experimental colitis. Neurogastroenterol. Motil..

[28] Philippe D., Dubuquoy L., Groux H., Brun V., Chuoi-Mariot M.T., Gaveriaux-Ruff C., Colombel J.F., Kieffer B.L., Desreumaux P. (2003). Anti-inflammatory properties of the mu opioid receptor support its use in the treatment of colon inflammation. J. Clin. Investig..

[29] Plein L.M., Rittner H.L. (2018). Opioids and the immune system—Friend or foe. Br. J. Pharmacol..

[30] Stein C., Kuchler S. (2013). Targeting inflammation and wound healing by opioids. Trends Pharmacol. Sci..

[31] Berg D.J., Zhang J., Weinstock J.V., Ismail H.F., Earle K.A., Alila H., Pamukcu R., Moore S., Lynch R.G. (2002). Rapid development of colitis in NSAID-treated IL-10-deficient mice. Gastroenterology.

[32] Hale L.P., Gottfried M.R., Swidsinski A. (2005). Piroxicam treatment of IL-10-deficient mice enhances colonic epithelial apoptosis and mucosal exposure to intestinal bacteria. Inflamm. Bowel Dis..

[33] Holgersen K., Kvist P.H., Markholst H., Hansen A.K., Holm T.L. (2014). Characterisation of enterocolitis in the piroxicam-accelerated interleukin-10 knock out mouse—A model mimicking inflammatory bowel disease. J Crohn’s Colitis.

[34] Graham D.B., Xavier R.J. (2020). Pathway paradigms revealed from the genetics of inflammatory bowel disease. Nature.

[35] Pigneur B., Escher J., Elawad M., Lima R., Buderus S., Kierkus J., Guariso G., Canioni D., Lambot K., Talbotec C. (2013). Phenotypic characterization of very early-onset IBD due to mutations in the IL10, IL10 receptor alpha or beta gene: A survey of the Genius Working Group. Inflamm. Bowel Dis..

[36] Begue B., Verdier J., Rieux-Laucat F., Goulet O., Morali A., Canioni D., Hugot J.P., Daussy C., Verkarre V., Pigneur B. (2011). Defective IL10 signaling defining a subgroup of patients with inflammatory bowel disease. Am. J. Gastroenterol..

[37] Kotlarz D., Beier R., Murugan D., Diestelhorst J., Jensen O., Boztug K., Pfeifer D., Kreipe H., Pfister E.D., Baumann U. (2012). Loss of interleukin-10 signaling and infantile inflammatory bowel disease: Implications for diagnosis and therapy. Gastroenterology.

[38] Madsen K.L., Malfair D., Gray D., Doyle J.S., Jewell L.D., Fedorak R.N. (1999). Interleukin-10 gene-deficient mice develop a primary intestinal permeability defect in response to enteric microflora. Inflamm. Bowel Dis..

[39] Holgersen K., Kvist P.H., Hansen A.K., Holm T.L. (2014). Predictive validity and immune cell involvement in the pathogenesis of piroxicam-accelerated colitis in interleukin-10 knockout mice. Int. Immunopharmacol..

[40] Rosen M.J., Karns R., Vallance J.E., Bezold R., Waddell A., Collins M.H., Haberman Y., Minar P., Baldassano R.N., Hyams J.S. (2017). Mucosal Expression of Type 2 and Type 17 Immune Response Genes Distinguishes Ulcerative Colitis From Colon-Only Crohn’s Disease in Treatment-Naive Pediatric Patients. Gastroenterology.

[41] Saruta M., Yu Q.T., Fleshner P.R., Mantel P.Y., Schmidt-Weber C.B., Banham A.H., Papadakis K.A. (2007). Characterization of FOXP3+CD4^+^ regulatory T cells in Crohn’s disease. Clin. Immunol..

[42] Rubtsov Y.P., Rasmussen J.P., Chi E.Y., Fontenot J., Castelli L., Ye X., Treuting P., Siewe L., Roers A., Henderson W.R. (2008). Regulatory T cell-derived interleukin-10 limits inflammation at environmental interfaces. Immunity.

[43] Sznurkowska K.L.J., Bryl E., Witkowski J.M., Hermann-Okoniewska B., Landowski P., Kosek M., Szlagatys-Sidorkiewicz A. (2020). Enhancement of Circulating and Intestinal T Regulatory Cells and Their Expression of Helios and Neuropilin-1 in Children with Inflammatory Bowel Disease. J. Inflamm. Res..

[44] Asseman C., Mauze S., Leach M.W., Coffman R.L., Powrie F. (1999). An essential role for interleukin 10 in the function of regulatory T cells that inhibit intestinal inflammation. J. Exp. Med..

[45] Basso L., Garnier L., Bessac A., Boue J., Blanpied C., Cenac N., Laffont S., Dietrich G. (2018). T-lymphocyte-derived enkephalins reduce Th1/Th17 colitis and associated pain in mice. J. Gastroenterol..

[46] Roy S., Wang J., Charboneau R., Loh H.H., Barke R.A. (2005). Morphine induces CD4^+^ T cell IL-4 expression through an adenylyl cyclase mechanism independent of the protein kinase A pathway. J. Immunol..

[47] Roy S., Wang J., Gupta S., Charboneau R., Loh H.H., Barke R.A. (2004). Chronic morphine treatment differentiates T helper cells to Th2 effector cells by modulating transcription factors GATA 3 and T-bet. J. Neuroimmunol..

[48] Sacerdote P., Manfredi B., Gaspani L., Panerai A.E. (2000). The opioid antagonist naloxone induces a shift from type 2 to type 1 cytokine pattern in BALB/cJ mice. Blood.

[49] Azarang A., Mahmoodi M., Rajabalian S., Shekari M.A., Nosratabadi J., Rezaei N. (2007). T-helper 1 and 2 serum cytokine assay in chronic opioid addicts. Eur. Cytokine Netw..

[50] Arrieta M.C., Madsen K., Doyle J., Meddings J. (2009). Reducing small intestinal permeability attenuates colitis in the IL10 gene-deficient mouse. Gut.

[51] Barreau F., Madre C., Meinzer U., Berrebi D., Dussaillant M., Merlin F., Eckmann L., Karin M., Sterkers G., Bonacorsi S. (2010). Nod2 regulates the host response towards microflora by modulating T cell function and epithelial permeability in mouse Peyer’s patches. Gut.

[52] Liu Z., Zhang P., Ma Y., Chen H., Zhou Y., Zhang M., Chu Z., Qin H. (2011). Lactobacillus plantarum prevents the development of colitis in IL-10-deficient mouse by reducing the intestinal permeability. Mol. Biol. Rep..

[53] Goldsmith J.R., Uronis J.M., Jobin C. (2011). Mu opioid signaling protects against acute murine intestinal injury in a manner involving Stat3 signaling. Am. J. Pathol..

[54] Valle L., Pol O., Puig M.M. (2001). Intestinal inflammation enhances the inhibitory effects of opioids on intestinal permeability in mice. J. Pharmacol. Exp. Ther..

[55] Valle L., Puig M.M., Pol O. (2000). Effects of mu-opioid receptor agonists on intestinal secretion and permeability during acute intestinal inflammation in mice. Eur. J. Pharmacol..

[56] Harari Y., Weisbrodt N.W., Moody F.G. (2000). Ileal mucosal response to bacterial toxin challenge. J. Trauma.

[57] Krystel-Whittemore M., Dileepan K.N., Wood J.G. (2016). Mast Cell: A Multi-Functional Master Cell. Front. Immunol..

[58] Harari Y., Weisbrodt N.W., Moody F.G. (2006). The effect of morphine on mast cell-mediated mucosal permeability. Surgery.

[59] Groschwitz K.R., Ahrens R., Osterfeld H., Gurish M.F., Han X., Abrink M., Finkelman F.D., Pejler G., Hogan S.P. (2009). Mast cells regulate homeostatic intestinal epithelial migration and barrier function by a chymase/Mcpt4-dependent mechanism. Proc. Natl. Acad. Sci. USA.

[60] Franchi S., Moretti S., Castelli M., Lattuada D., Scavullo C., Panerai A.E., Sacerdote P. (2012). Mu opioid receptor activation modulates Toll like receptor 4 in murine macrophages. Brain Behav. Immun..

[61] Moller P., Koretz K., Leithauser F., Bruderlein S., Henne C., Quentmeier A., Krammer P.H. (1994). Expression of APO-1 (CD95), a member of the NGF/TNF receptor superfamily, in normal and neoplastic colon epithelium. Int. J. Cancer.

[62] Park S.M., Chen L., Zhang M., Ashton-Rickardt P., Turner J.R., Peter M.E. (2010). CD95 is cytoprotective for intestinal epithelial cells in colitis. Inflamm. Bowel Dis..

[63] Jaume M., Jacquet S., Cavailles P., Mace G., Stephan L., Blanpied C., Demur C., Brousset P., Dietrich G. (2004). Opioid receptor blockade reduces Fas-induced hepatitis in mice. Hepatology.

[64] Wang J., Charboneau R., Barke R.A., Loh H.H., Roy S. (2002). Mu-opioid receptor mediates chronic restraint stress-induced lymphocyte apoptosis. J. Immunol..

[65] Yin D., Tuthill D., Mufson R.A., Shi Y. (2000). Chronic restraint stress promotes lymphocyte apoptosis by modulating CD95 expression. J. Exp. Med..

[66] Jaume M., Laffont S., Chapey E., Blanpied C., Dietrich G. (2007). Opioid receptor blockade increases the number of lymphocytes without altering T cell response in draining lymph nodes in vivo. J. Neuroimmunol..

[67] Benard A., Cavailles P., Boue J., Chapey E., Bayry J., Blanpied C., Meyer N., Lamant L., Kaveri S.V., Brousset P. (2010). μ-Opioid receptor is induced by IL-13 within lymph nodes from patients with Sezary syndrome. J. Investig. Dermatol..

[68] Jung C., Meinzer U., Montcuquet N., Thachil E., Chateau D., Thiebaut R., Roy M., Alnabhani Z., Berrebi D., Dussaillant M. (2012). Yersinia pseudotuberculosis disrupts intestinal barrier integrity through hematopoietic TLR-2 signaling. J. Clin. Investig..

[69] Le Faouder P., Baillif V., Spreadbury I., Motta J.P., Rousset P., Chene G., Guigne C., Terce F., Vanner S., Vergnolle N. (2013). LC-MS/MS method for rapid and concomitant quantification of pro-inflammatory and pro-resolving polyunsaturated fatty acid metabolites. J. Chromatogr. B.

[70] Pujo J., Petitfils C., Le Faouder P., Eeckhaut V., Payros G., Maurel S., Perez-Berezo T., Van Hul M., Barreau F., Blanpied C. (2020). Bacteria-derived long chain fatty acid exhibits anti-inflammatory properties in colitis. Gut.

