# Supplementary material for: Peripheral Opioid Receptor Blockade Enhances Epithelial Damage in Piroxicam-Accelerated Colitis in IL-10-Deficient Mice
Source: Int J Mol Sci. 2021 Jul 9;22(14):7387. doi: 10.3390/ijms22147387 (PMC8304158; doi:10.3390/ijms22147387)
Supplement: Supplementary file 1 [file ijms-22-07387-s001.zip › ijms-1255686-SI.pdf]

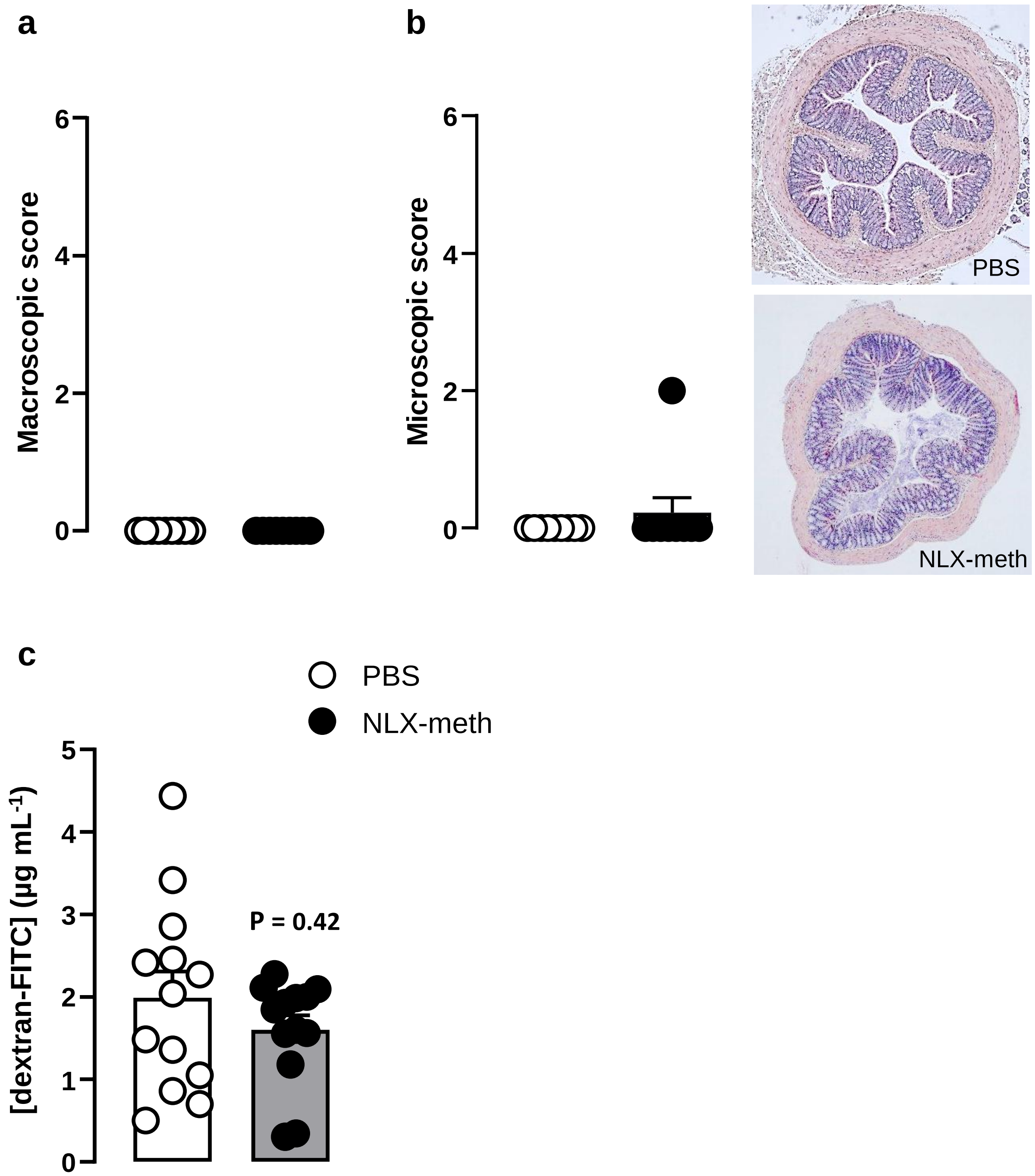

Figure S1. C57BL/6 mice were injected with either PBS (white symbols) or naloxone-methiodide (NLX-meth) (black symbols) two days apart for ten days (n = 13 / group). Macroscopic (a) and microscopic (b) colonic tissue damage as well as paracellular permeability (expressed as the mean of FITC-dextran concentration in serum (µg mL^{-1}) 4 hours after gavage with dextran) (c) were similar in mice treated or not with NLX-meth. Data are expressed as mean ± SEM. Statistical analyses were performed using Mann-Whitney U test.
